# Intravascular papillary endothelial hyperplasia of the finger: a case of Masson’s tumor

**DOI:** 10.1080/23320885.2021.1884560

**Published:** 2021-02-15

**Authors:** Kun-Yong Sung, Seungkoo Lee, Yeonjin Jeong, Sang-Yeul Lee

**Affiliations:** a Department of Plastic and Reconstructive Surgery, School of Medicine, Kangwon National University, Chuncheon, Korea; b Department of Anatomic Pathology, School of Medicine, Kangwon National University, Chuncheon, Korea; c Department of Plastic and Reconstructive Surgery, Kangwon National University Hospital, Chuncheon, Korea

**Keywords:** Blood vessels, endothelium, fingers, cysts, hyperplasia

## Abstract

Intravascular papillary endothelial hyperplasia is an uncommon benign vascular lesion characterized by a reactive proliferation of endothelial cells. The lesion of the finger often presents diagnostic challenges to surgeons because of its rarity. We report a case of intravascular papillary endothelial hyperplasia to facilitate the recognition of this uncommon lesion.

## Introduction

Intravascular papillary endothelial hyperplasia (IPEH), also known as Masson’s tumor, is an uncommon benign vascular lesion characterized by a reactive proliferation of endothelial cells. The lesion arising from an ulcerated hemorrhoidal vein was first described by Masson [1] as a ‘vegetant intravascular hemangioendothelioma’. Thereafter, it has been reported with diverse descriptions such as intravascular angiomatosis, intravenous vascular proliferation, Masson’s pseudoangiosarcoma, and Masson’s tumor. Masson believed that it was a specific type of hemangioma. However, Henschen [2] proposed that the endothelial proliferation is reactive rather than neoplastic. The descriptive term ‘intravascular papillary endothelial hyperplasia’ was first proposed by Clerkin and Enzinger [3].

IPEH usually presents as a well-defined, superficial papule or a deep nodule in the skin or subcutaneous tissue. It commonly occurs in the head, neck, and upper extremities in the third and fourth decades of life. Less frequently, it also occurs in the adrenal gland, heart, intestine, kidney, liver, lungs, popliteal artery, and superior vena cava. IPEH of the finger often poses a diagnostic challenge for hand surgeons because of its rarity and absence of characteristic clinical features. Herein, we report a case of IPEH manifesting as a cystic nodule in the finger to facilitate the recognition of this uncommon lesion. We also present other conditions to be included in the differential diagnosis of finger nodules through a comprehensive review of the relevant literature.

## Case report

**Figure 1.**
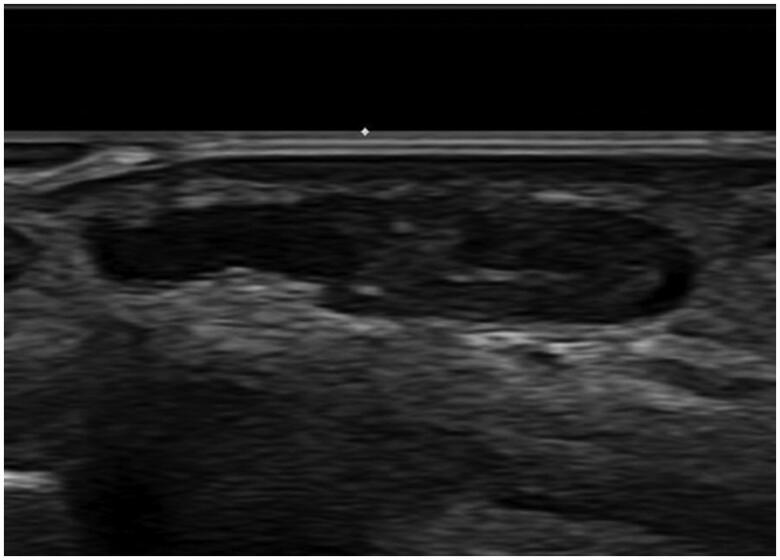
Preoperative ultrasonography reveals about 0.7 × 0.3 × 1.2 cm sized cystic lesion with no remarkable increased vascularity.

A 53-year-old woman presented with a small, asymptomatic, protruding mass on the volar surface of the proximal phalanx of the left index finger. She noticed the lesion 3 months prior to her visit to our clinic. She had no remarkable medical or trauma history. On clinical examination, the lesion revealed a subcutaneous cystic nodule with no discoloration of the overlying skin. It was non-tender and movable. Preoperative ultrasonography revealed a cystic lesion approximately 0.7 × 0.3 × 1.2 cm in size, with no remarkable increase in the vascularity. Ultrasonography was ultimately suggestive of a probable ganglion cyst (Figure 1).

**Figure 2.**
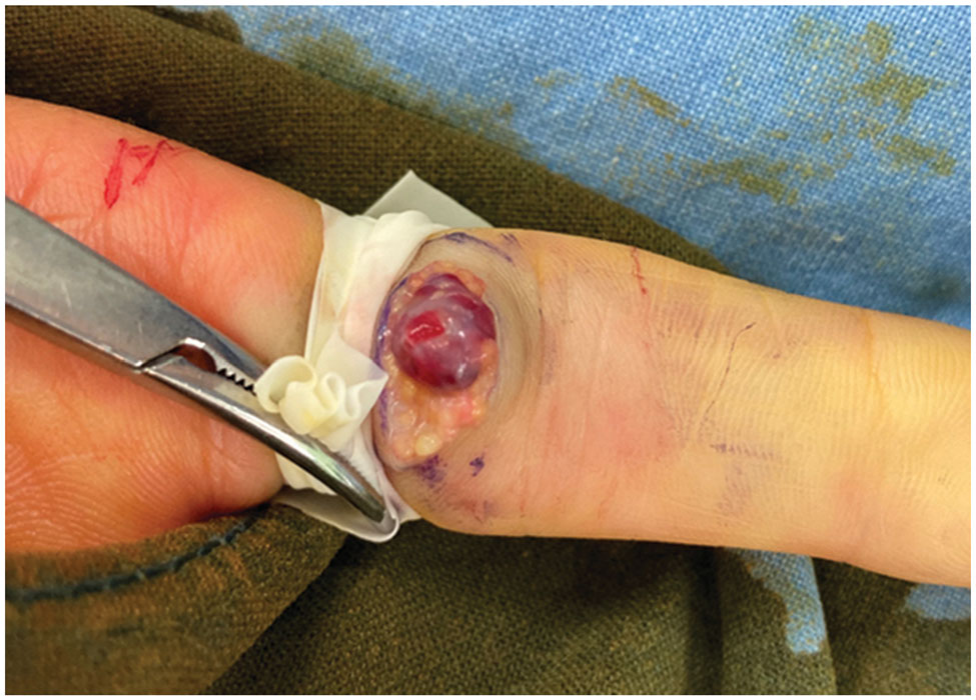
Intraoperative photograph shows a soft, red purple, and well-circumscribed cystic mass looking like a hemangioma at the volar surface of left 2nd proximal phalanx.

**Figure 3.**
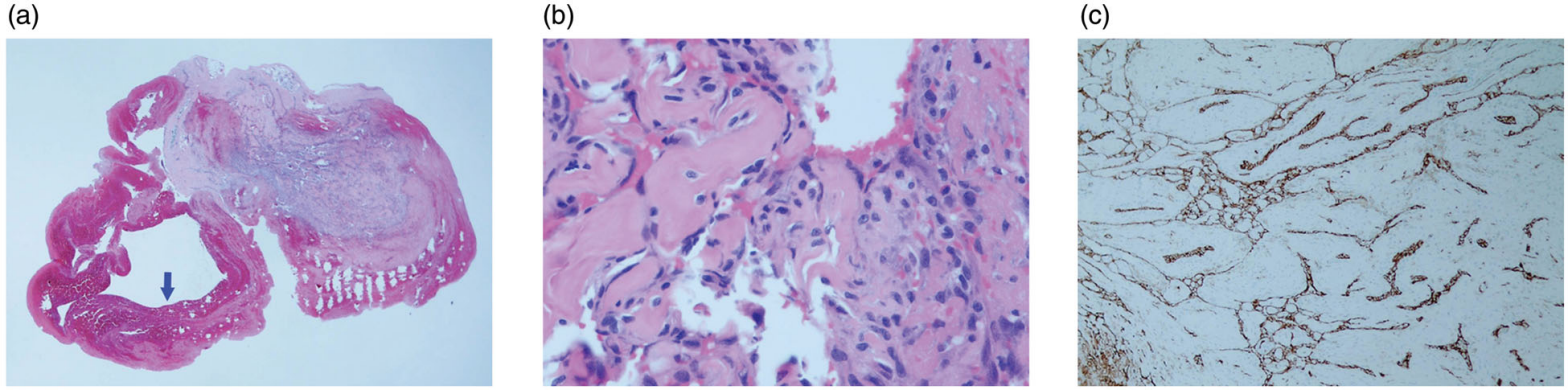
Histopathologic appearance of the lesion. (a) The specimen showed intravascular proliferative lesion with organizing thrombus (arrow) (H&E, ×15). (b) High magnification of papillary proliferations revealed fibrinous and hyaline stalks lined by endothelial cells (H&E, ×400). (c) These lining cells were positive for anti-CD31 (immunohistochemical stain, clone JC70, Cell Marque, ×100).

The lesion was excised under digital nerve block. It was located in the subcutaneous plane and easily dissected from the surrounding tissues. Intraoperatively, gross examination of the tumor showed a soft, red purple, and well-circumscribed cystic mass, which looked like a hemangioma (Figure 2); however, remarkable feeding vessels were not observed while dissecting the lesion. Histologic examination revealed intravascular arborizing endothelial tissue with an organizing thrombus (Figure 3(a)). The vascular tissue showed intravascular endothelial cell proliferation with characteristic papillary hyaline cores (Figure 3(b)). On immunohistochemical analysis, the endothelial cells were positive for the endothelial markers CD31 and CD34 (Figure 3(c)). Based on these histopathological findings, the patient was diagnosed as having IPEH.

## Discussion

IPEH is known to be a non-neoplastic reactive endothelial proliferation. These lesions account for approximately 2% of the vascular tumors of skin and soft tissue [4]. Some of them present with tenderness or discoloration of the overlying skin. The lesions are usually solitary, but multiple lesions have occasionally been reported.

The pathogenesis of IPEH is poorly understood. Most evidence suggests that it is a reactive process that occurs in the setting of unusual thrombus organization. However, the order of disease progression remains controversial. Either endothelial proliferation is the primary process with secondary thrombus formation [1] or the thrombus serves as a matrix for papillary proliferation [5]. Several investigators have described that this reactive process could be related to an antecedent trauma history and the consecutively formed thrombus [3]. Levere et al. proposed that IPEH could be initially triggered by the release of basic fibroblast growth factor (bFGF) from macrophages recruited to the area by minor trauma or irritation. This release of bFGF could stimulate the proliferation of endothelial cells, which in turn would secrete more bFGF, which would then set up a positive feedback loop causing a cascade of endothelial proliferation [6].

Three types of IPEH are recognized [7]. The primary, pure, or intravascular type (56%) occurs in dilated vascular spaces. The mass is usually a small subcutaneous lesion and occurs most commonly in the finger. The secondary or mixed type (40%) occurs in pre-existing vascular lesions such as hemangiomas, pyogenic granulomas, or vascular malformations. About half of the mixed type lesions were intramuscular. The extravascular type (4%) arises from a hematoma. The extravascular type lesions tend to be larger than the other types.

The characteristic histological feature of IPEH is the presence of papillary structures covered with hyperplastic endothelial cells within the vascular lumen. Besides the intra-luminal location of the lesion, other useful diagnostic features include an intimate association with the organizing thrombus, and the absence of necrosis, cellular pleomorphism, and mitotic activity. IPEH needs to be differentiated from angiosarcoma as the lesion may clinically and histologically be mistaken for a low grade angiosarcoma [8]. A critical distinction between angiosarcoma and IPEH is that angiosarcomas are typically not present inside the lumen of blood vessels [9]. Moreover, unlike angiosarcoma, the endothelial cells of IPEH lack necrosis, marked pleomorphism, significant mitotic activity, and solid sheet formation.

Apart from angiosarcoma, other conditions that can be included in the differential diagnosis of finger nodules are: giant cell tumor of the tendon sheath, digital mucous cyst/digital ganglion cyst/digital myxoid pseudocyst, glomus tumor, neurogenic tumor, dermatofibroma, rheumatoid nodules, subcutaneous sarcoidosis, vascular malformations, angioleiomyoma, Dabska tumor, Heberden Node, Bouchard node, and gouty tophus (Table 1). Ultrasonography, computed tomography and magnetic resonance imaging are often helpful in differentiating these subcutaneous nodules. However, confirmative diagnosis is usually made after microscopic examination.

**Table 1.** Differential diagnosis for subcutaneous finger nodules.

Conditions	Clinical features	Histologic features
Giant cell tumor of the tendon sheath	The second most common benign neoplasm of the hand/painless slow growing nodule/ occasional recurrence after excision	A circumscribed nodule comprised of a population of oval cells with eosinophilic fibrous stroma and scattered multinucleated giant cells
Digital mucous cyst (Digital ganglion cyst)	A solitary, dome-shaped, cystic nodule on the dorsum of the fingers and rarely on the toes, usually involving the base of the nail	Myxomatous type: a large cystic space containing mucin with no lining of the cyst wall/ganglionic type: a cystic space with a well-defined fibrous wall
Glomus tumor	A small, blue-red nodule/occurs on the extremities, especially in the subungual area of the digits, palm, wrist, forearm, or foot	Irregularly shaped capillary sized vessels with compact nests of uniformly round to ovoid glomus cells set in a hyalinized or myxoid stroma
Neurofibroma	Skin-colored, dome-shaped nodules with a pendulous or plexiform morphology	A mix of Schwann cells, perineurial cells/interspersed with nerve fibers, wavy collagenous strands, and myxoid matrix
Digital nerve schwannoma	A benign nerve sheath tumor originating from Schwann cells	Biphasic tumor with Antoni A and Antoni B component.
Dermatofibroma	A firm subcutaneous benign nodule/mostly in the lower legs	Benign spindle cell tumor with histiocytoid cell features.
Subcutaneous sarcoidosis	Multiple painless, firm, mobile nodules located on the extremities	Inflammatory and granulomatous reactions with inflammatory cells
Arteriovenous malformation	Slow flow: capillary, venous, lymphatic malformation, fast flow: arteriovenous malformation	Abnormally formed channels within a vascular apparatus that are lined by endothelial cells and do not undergo abnormal cellular turnover
Angioleiomyoma	A solitary, slow growing nodule originating from smooth muscle cells	Benign smooth muscle cells with vascular tissue without elastic lamina tissue
Epithelioid Hemangioendothelioma	Intermediate grade vascular malignancies/commonly affect the soft tissues, liver, lungs and bones	Large epithelioid perivascular cells with eosinophilic cytoplasm and ‘blister cells’
Dabska Tumor	A rare, low-grade angiosarcoma that often affects the skin and subcutaneous tissues	Anastomosing vascular channels, some of which contain papillary projections or tuft-like structures
Rheumatoid nodules	Associated with rheumatoid arthritis lesion/ firm nodules	A shell of fibrous tissue with fibrinoid necrosis/cellular palisading.
Heberden Node	A bony swelling of the DIP joint/ formation of osteophytes/ a sign of osteoarthritis	
Bouchard node	A bony outgrowth or gelatinous cyst on the PIP joints/ associated with severe arthritis osteoarthritis	
Gouty tophus	Usually painless/bumps made of urate crystals/cardinal feature of advanced gout	Large aggregates of urate crystals, granulomatous inflammation

The preferred treatment of IPEH is marginal excision. Additional tissue removal is usually not required to prevent recurrence. Its prognosis is good because it does not transform to a malignant neoplasm and does not recur after proper management. However, a thorough understanding of this lesion is needed to differentiate it from angiosarcomas as well as other tumors causing finger nodules and to ensure proper management.
